# Apoptosis Induced by *Ginkgo biloba* (EGb761) in Melanoma Cells Is Mcl-1-Dependent

**DOI:** 10.1371/journal.pone.0124812

**Published:** 2015-04-10

**Authors:** Yufang Wang, Junping Lv, Yao Cheng, Jipei Du, Degao Chen, Chengtao Li, Ji Zhang

**Affiliations:** 1 Department of Pathophysiology, School of Preclinical and Forensic Medicine, Sichuan University, Chengdu, P.R. China; 2 Department of Pharmacology, Beijing Institute of Biomedicine, Beijing, P.R. China; 3 Shanghai Key Laboratory of Forensic Medicine, Institute of Forensic Sciences, Ministry of Justice, Shanghai, P.R. China; 4 Department of Forensic Genetics, School of Preclinical and Forensic Medicine, Sichuan University, Chengdu, P.R. China; Columbia University, UNITED STATES

## Abstract

Melanoma is an aggressive skin cancer. Unfortunately, there is currently no chemotherapeutic agent available to significantly prolong the survival of the most patients with metastatic melanomas. Here we report that the *Ginkgo biloba* extract (EGb761), one of the most widely sold herbal supplements in the world, potently induces apoptosis in human melanoma cells by disturbing the balance between pro- and anti-apoptosis Bcl-2 family proteins. Treatment with EGb761 induced varying degrees of apoptosis in melanoma cell lines but not in melanocytes. Induction of apoptosis was caspase-dependent and appeared to be mediated by the mitochondrial pathway, in that it was associated with reduction in mitochondrial membrane potential and activation of Bax and Bak. Although EGb761 did not cause significant change in the expression levels of the BH3-only Bcl-2 family proteins Bim, Puma, Noxa, and Bad, it significantly downregulated Mcl-1 in sensitive but not resistant melanoma cells, suggesting a major role of Mcl-1 in regulating apoptosis of melanoma cells induced by EGb761. Indeed, siRNA knockdown of Mcl-1 enhanced EGb761-induced apoptosis, which was associated with increased activation of Bax and Bak. Taken together, these results demonstrate that EGb761 kills melanoma cells through the mitochondrial apoptotic pathway, and that Mcl-1 is a major regulator of sensitivity of melanoma cells to apoptosis induced by EGb761. Therefore, EGb761 with or without in combination with targeting Mcl-1 may be a useful strategy in the treatment of melanoma.

## Introduction

The incidence of melanoma continues to rise in many countries, and it has become one of the main causes of cancer-related morbidity and mortality all over the world [1]. Surgery is the most effective treatment of melanoma at early stages. Unfortunately, there is currently no curative therapy once the disease spreads beyond the primary site. Therefore, treatment of metastatic melanomas continues to pose a therapeutic challenge [2]. This is closely related to resistance of melanoma cells to apoptosis induced by conventional chemotherapeutics as well as other biological agents [3–5]. Understanding of mechanisms responsible for the resistance is critical for identification of new therapeutic targets and development of novel treatment approaches [6–8].

Many chemotherapeutic drugs kill cancer cells by inducing apoptosis [9–11]. This is commonly mediated by the mitochondria apoptotic pathway that is regulated by Bcl-2 family proteins [12–14]. Bcl-2 family members have either pro- or anti-apoptotic activities, and regulate the mitochondrial apoptotic pathway by controlling permeabilization of the outer mitochondrial membrane. Anti-apoptotic proteins such as Bcl-2, Bcl-X_L_, and Mcl-1 protect mitochondrial integrity, whereas activation of pro-apoptotic proteins of the family promotes the release of mitochondrial proteins, such as cytochrome c, Smac/DIABLO, and AIF into the cytosol [15,16]. This eventually leads to cell death by apoptosis. Pro-apoptotic Bcl-2 family proteins can be further divided into two groups, the BH3-only proteins including Bid, Bad, Bim, Puma, and Noxa, and their effectors, the multi-domain proteins Bax and Bak. BH3-only proteins act as upstream sentinels of cellular damage and derangement [17]. Upon activation, they activate Bax and/or Bak by binding directly to them or by displacing them indirectly from anti-apoptotic Bcl-2 family members. As an anti-apoptotic Bcl-2 family protein, Mcl-1 has been proposed to play a unique apical role in protection of cells against apoptosis by neutralizing both Bax and Bak [18]. Indeed, elimination of Mcl-1 is required at an early stage of induction of apoptosis [19].

*Ginkgo biloba* has long been thought to have medicinal properties. Its extracts are among the most widely sold herbal supplements in the world. The *Ginkgo biloba* extract EGb761 is a standard extract containing 24% ginkgo lavone glycoside and 6% terpene lactones and is considered as a polyvalent therapeutic agent in the treatment of various diseases [20]. For example, it is widely used for the treatment of several neurological disorders for its anti-oxidant and anti-platelet properties [21–23]. EGb761 has anti-oxidant effects in cerebral and peripheral arterial diseases by inhibiting ROS generation [24]. Moreover, it has been recently reported that EGb761 has anti-proliferation and apoptosis-inducting effects in various cancers such as those of the pancreas and colon [25–27]. However, little is known about the potential effect of EGb761 on human melanoma. In this study, we have examined the response of melanoma cells to treatment with EGb761. We report here that EGb761 triggers caspase-dependent apoptosis of melanoma cells through the mitochondrial apoptotic pathway. Moreover, we show that the anti-apoptotic Bcl-2 family protein Mcl-1 plays an important role in regulation of sensitivity of melanoma cells to apoptosis induced by EGb761.

## Materials and Methods

### Cell culture and reagents

Human melanoma cell lines Mel4405, IgR3, Mel-AT, Mel-RMu, Mel-RM, Mel-CV, Sk-Mel-28, Sk-Mel-110, Mel-1007 and MM200 were kindly provided by Dr. Xu Dong Zhang (University of Newcastle, New South Wales, Australia.) and were cultured in DMEM containing 5% FCS (Commonwealth Serum Laboratories)[28,29]. Primary Human Melanocyte was also a gift from Dr. Xu Dong Zhang and cultured in Adult Melanocyte Growth Kit (ATCC PCS-200-042). The caspase inhibitor z-VAD-fmk, z-LEHD-fmk, and z-DEVD-fmk were purchased from Calbiochem. Anti-Bax Antibody (6A7) (MA5-14003) was purchased from Thermo Scientific and anti-Bak Antibody (04–433) from EMD Millipore. The rabbit polyclonal Abs against caspase-3, caspase-8, and caspase-9 were from Stressgen. The mouse MAbs against Bcl-2, Bcl-X_L_, Mcl-1, and Bad were purchased from Santa Cruz Biotechnology. The rabbit polyclonal Abs against PUMA, and Bid were from Cell Signaling Technology. The MAbs against Noxa and the polyclonal Ab against Bim were purchased from Imgenex. The mouse MAb against PARP was from Pharmingen (Bioclone). EGb761, a standardized Ginkgo biloba extract, were purchased from Schwarz Pharma AG (Monheim, Germany) and solubilized by 5% ethanol freshly for cells treatment.

### Cell viability assay

Cell viability was assessed by 3-(4, 5-dimethylthylthiazol- 2-yl)-2, 5-diphenyltetra-zolium bromide (MTT) dye reduction assay. Melanoma cells were seeded at a concentration of 5×10^3^ cells/well in a 96-well plate. The dosage of EGb761 used in this study was initially determined by a calculation with its clinical dosage, using formula of “concentration for *in vitro* use (μg/ml) = (50D/5000)/50%×10^3^ (D: dosage in clinic using: mg.kg^-^/day, 50 stands for the average body weight(kg) of an adult; 5000 is the whole volume of body blood(ml); 50% means median lethal dose also known as LD50 and 10^3^ converts mg to μg)[17,30,31]. At the end of the treatment, 5mg/ml MTT was added and the cells were incubated for another 4 h. Cell viability was detected by measuring the absorbance at 490 nm wavelength using Bio-rad microplate reader. Cell viability (%) = the absorbance of experimental group/the absorbance of blank control group × 100%.

### Apoptosis assay

Quantitation of apoptotic cells by measurement of sub-G1 DNA content using the propidium iodide (PI) method or by PI and Annexin-V staining was carried out as described elsewhere [16,20]. Briefly, cells were plated at 4×10^5^ cells/well in six-well plates and treated with EGb761 or 5% ethanol (control) in DMEM containing 5% FCS. After treatment, cells were stained with Annexin V-FITC and propidium iodide (PI) in binding buffer using a Roche Annexin V-FLUOS Staining kit (Roche, Cat.No.11858777001) according to the manufacturer’s protocol. Cells were examined by flow cytometry (FACSort, Becton Dickson). Annexin V-positive/PI-positive and Annexin V-positive/ PI-negative cell populations were defined as the apoptotic population among total gated cells.

### Measurement of conformational change of Bax and Bak by flow cytometry

Measurement of conformational change of Bax and Bak was performed essentially as described [32]. After treatment with EGb761 or 5% ethanol, cells were washed with PBS and fixed in 1 ml PBS/0.5% paraformaldehyde (v/v) on ice for 30 min and washed in PBS/1% FCS. Staining with conformation specific anti-Bax NT antibody or anti-Bak was performed by incubating cells in 50 μl staining buffer (PBS,1% FCS, 0.1% saponin) containing 0.1 mg of the respective antibody on ice for 30 min. Then, cells were washed in staining buffer, resuspended in 50 μl staining buffer containing 0.1 mg fluorescein-labelled F(ab')2 goat anti-rabbit IgG (H+L) anti-serum and incubated on ice for 30 min in the dark. After washing in PBS with 1% FCS, intracellular staining of Bax was immediately measured with Becton Dickinson FACScan flow cytometer.

### Small RNA interference (siRNA)

Melanoma cells were seeded at 1×10^5^ cells per well in 24-well plates and allowed to reach 50% confluence on the day of transfection. The small interfering RNA (siRNA) constructs used were obtained as the siGENOMESMARTpool reagents (Dharmacon), the siGENOMESMARTpool Mcl-1 (M-004501-04-0010). The non-targeting siRNA control, SiConTRol- Non-targeting SiRNA pool (D-001206-13-20) was also obtained from Dharmacon. Cells were transfected with 50 to 100 nmol/L siRNA in Opti-MEM medium (Invitrogen) using LipofectAMINE reagent (Invitrogen) according to the manufacturer’s transfection protocol. Twenty-four hours after transfection, the cells were treated with EGb761 at 400 μg/ml for another 48 h before quantitation of apoptotic cells by measurement of percentage of apoptosis in flow cytometry. Efficiency of siRNA was measured by Western blot analysis.

### Mitochondrial membrane potential (ΔΨm)

Cells were seeded at 1×10^5^ cells/well in 24-well plates, and allowed to reach exponential growth for 24 h before treatment. Changes in ΔΨm were studied by staining the cells with the cationic dye, JC-1(5, 5’, 6, 6’-tetrachloro-1, 1’, 3, 3’- tetraethyl-benzamidazolocarbocyanin iodide) (Molecular Probes, T3168), according to the manufacturer’s instructions as described previously [33].

### Preparation of mitochondrial and cytosolic fractions

Cells were washed in buffer A (100 mM sucrose, 1 mM EGTA, 20 mM MOPS, pH 7.4) and resuspended in buffer B (buffer A plus 5% Percoll, 0.01% digitonin and a cocktail of protease inhibitors). After 15 min incubation on ice, unbroken cells and nuclei were pelleted by centrifugation at 2500 g for 10 min. The supernatant was centrifuged at 15 000 g for 15 min to pellet mitochondria which were resuspended in buffer C (300 mM sucrose, 1 mM EGTA, 20 mM MOPS pH 7.4 and the cocktail of protease inhibitors). The supernatant was centrifuged further at 100 000g for 1 h. The resultant supernatant and the pellet were designated as the cytosolic and microsomal fractions, respectively [34].

### Western blot analysis

Cells treated with EGb761 were lyzed with RIPA buffer with proteinase inhibitors and denatured by boiling with sample buffer. For separation of proteins, 30 μg sample was loaded in each lane. After electrophoresis, proteins were transferred to PVDF membrane. Membranes were blocked with 5% non-fat dry milk in TBST and then incubated with specific Ab in blocking buffer (TBST+1%milk) and HRP-conjugated secondary Ab. Reacted Ab was detected by Amersham ECL Select Western Blot Kit (GE). The images were captured with ImageQuant LAS (GE). The intensity of bands was quantified relative to corresponding GAPDH bands with ImageQuant TL software v7.0 [35].

### RNA isolation and real-time RT-PCR

Total RNA was isolated using the Trizol method (Invitrogen). A total of 4 μg of RNA were reverse-transcribed using the TaqMan Reverse Transcription Reagents Kit (Applied Biosystems; N8080234) and amplified using Power SYBR Green Master Mix (Applied Biosystems; 4367659) on the 7300 Real-Time PCR system (Applied Biosystems). Mcl-1 specific primers (5’-ATGCTTCGGAAACTGGACAT-3’ and 5’-TCCTGATGCCACCTTCTAGG-3’) and actin-specific primers (5’-AGAAAATCTGGCACCACACC-3”and 5’-AGAGGCG-TACAGGGATAGCA-3’) amplified fragments of the full-length transcripts. Results were normalized to actin.

### Statistical analysis

Two-tailed student’s *t*-test was used to assess differences in values between experimental groups. A *P*-value of <0.05 was considered to be statistically significance.

## Results

### EGb761 induces apoptosis of melanoma cells

**Fig 1:**
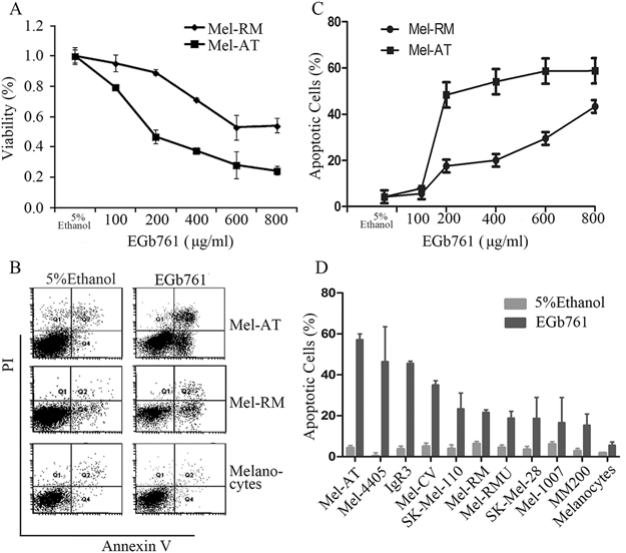
EGb761 inhibits cell growth and induces apoptosis in melanoma cells in vitro. (A) Effects of EGb761 on proliferation in Mel-RM and Mel-AT cells. After EGb761 treatment at indicated concentration (100-800ug/ml) or 5% ethanol treatment as the vehicle control, Mel-RM and Mel-AT cells proliferation was assessed in a 96-well plate at 72 hours by MTT assay. The data shown are the mean ± SEM of three individual experiments. (B) Representative dot plots showing fluorescence channel analyses of melanoma cells after dual staining with Annexin V and propidium iodide. Melanocytes, Mel-RM and Mel-AT cells were treated with EGb761 at 400 μg/ml for 24h (5% ethanol treatment as the vehicle control) and then stained with FITC-conjugated Annexin V (green fluorescence, horizontal axis) and PI(red fluorescence, vertical axis), and analyzed using flow cytometry. The data shown are representative of three individual experiments. (C) EGb761 induces apoptosis of melanoma cells. Mel-RM and Mel-AT cells treated with EGb761 (100–800 μg/ml) or 5% ethanol treatment as the vehicle control for indicated intervals were subjected to measurement of apoptosis by the Annexin V/PI staining method using flow cytometry. The data shown are the mean ± SEM of three individual experiments. (D) Induction of apoptosis by EGb761 in a panel of melanoma cell lines and melanocytes. Cells treated with EGb761 (400 μg/ml) or 5% ethanol for 24h were subjected to measurement of sub-G1content by the propidiumiodide method using flow cytometry. Columns, mean of three individual experiments; Bars, SEM.

To examine the potential effect of EGb761 on melanoma cell proliferation and survival, we treated Mel-RM and Mel-AT cells with EGb761 at increasing concentrations ranging from 100 to 800 μg/ml, or the vehicle control (5% ethanol). As shown in Fig 1A, EGb761 inhibited viability of Mel-AT, and to a lesser extent, viability of Mel-RM cells as measured using MTT assays by 72 hours after treatment. EGb761 at 400 μg/ml that is a clinically achievable concentration [17,30,31]reduced viability of Mel-AT and Mel-RM by 72% and 38%, respectively. Inhibition of viability of melanoma cells by EGb761 appeared largely due to induction of apoptosis as shown by quantitation of apoptotic cells using dual staining with Annexin V and PI (Fig 1B and 1C). Induction of apoptosis by EGb761 was confirmed in a panel of melanoma cell lines (Fig 1D). However, there were wide variations in sensitivity of the melanoma cell lines to EGb761-induced apoptosis, which was not associated with the BRAF^V600E^ mutation (Fig 1D), the most common driver mutation in melanoma cells [36]. For example, Mel-RM and Mel-AT cells that carried the wild-type BRAF had different sensitivity to EGb761-induced apoptosis, whereas MM200 and Mel-RMu that harbored the BRAF^V600E^ were relatively resistant to apoptosis induced by EGb761. Of note, EGb761 did not induce significant apoptosis in melanocytes (Fig 1B and 1D).

### Apoptosis of melanoma cells induced by EGb761 is caspase-dependent

**Fig 2:**
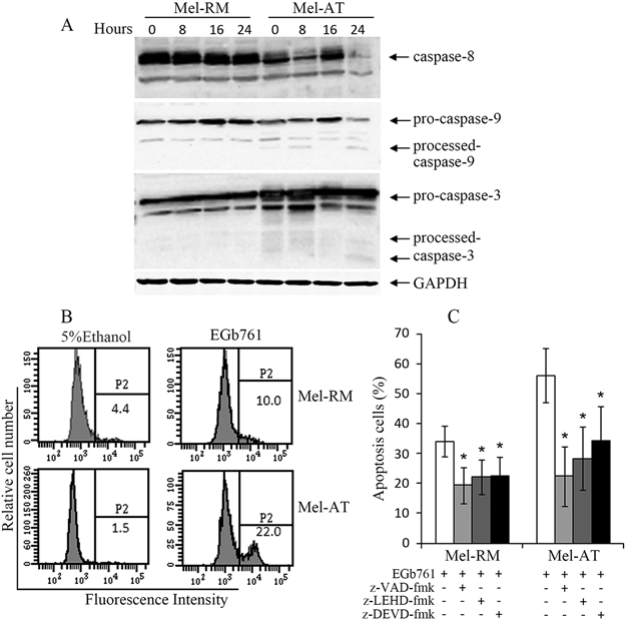
EGb761 induces partially Caspase-Dependent Apoptosis in Melanoma Cells. (A) EGb761 induces caspase activation. Whole cell lysates from Mel-RM and Mel-AT cells treated with EGb761 (400 μg/ml) for the indicated time periods were subjected to Western blot analysis. The data shown are representative of three individual experiments. (B) Mel-RM and Mel-AT cells with or without treatment with EGb761 (400 μg/ml) for 16 h were subjected to flow cytometry analysis of caspase-3 activation using an antibody that specifically recognizes the activated form of caspase-3. The data shown are representative of three individual experiments. (C) Mel-RM and Mel-AT cells were pretreated with the pan-caspase inhibitor z-VAD-fmk (20 μmol/L), the caspase-3 inhibitor z-DEVD-fmk (30 μmol/L), or the caspase-9 inhibitor z-LEHD-fmk (30 μmol/L) 1h before adding EGb761 (400 μg/ml) for another 24h, respectively. Apoptosis was measured by the propidium iodide method using flow cytometry. Data are the mean ± SEM of three individual experiments. * Present p<0.05 vs control.

Induction of apoptosis by either extrinsic or intrinsic apoptotic pathways is largely caspase-dependent [37]. To determine the mechanism responsible for EGb761-induced apoptosis, we analyzed activation of the caspase cascade in melanoma cells after treatment with EGb761 by Western blotting. Fig 2A shows that, activation of caspase-9 was evident in Mel-AT cells as early as 8 hours after treatment as indicated by the appearance of the p37 form of cleaved caspase-9, whereas activation of caspase-3 was detected at 24h as evidence by the appearance of p17 form of cleaved caspase3. In contrast, little or no caspase-3 and -9 activation was detected in Mel-RM cells treated with EGb761 (Fig 2A). Activation of caspase-8 was not detected in both Mel-AT and Mel-RM cells (Fig 2A). The difference in activation of caspase-3 between Mel-RM and Mel-AT cells induced by EGb761 was confirmed using flow cytometry with a monoclonal antibody that specifically recognizes the active form of caspase-3 (Fig 2B) [35].

To further confirm the role of caspases in EGb761-induced apoptosis, Mel-RM and Mel-AT cells were pretreated with the general inhibitor, z-VAD-fmk (20 μmol/L), the caspase-3 inhibitor, z-DEVD-fmk (30 μmol/L), and the caspase-9 inhibitor, z-LEHD-fmk (30 μmol/L) 1 hour before the addition of EGb761 (400 μg/mL) for another 24h. As shown in Fig 2C, z-VAD-fmk significantly inhibited EGb761-induced apoptosis in both Mel-RM and Mel-AT cells. Similarly, the caspase-9 and casapse-3 inhibitors also inhibited apoptosis induced by EGb761. These results indicate that apoptosis of melanoma cells induced by EGb761 is dependent on the caspase-9 and caspase-3 activation, and suggest that the mitochondrial apoptotic pathway plays an important role in induction of apoptosis of melanoma cells by EGb761.

### Apoptosis of melanoma induced by EGb761 involves activation of Bax and Bak

**Fig 3:**
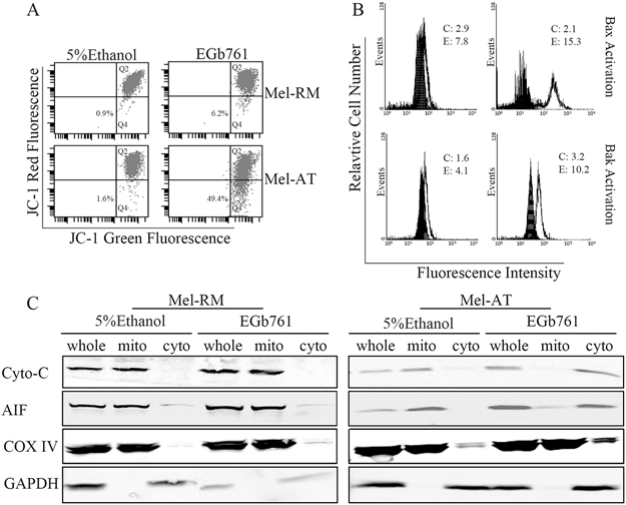
Apoptosis of melanoma induced by EGb761 involves activation of Bax and Bak and changes in mitochondria. (A) EGb761 induces reduction in ΔΨm. Mel-RM and Mel-AT cells treated with EGb761 (400 μg/ml) for the indicated intervals were subjected to measurement of ΔΨm by JC-1staining in flow cytometry. The number in each left bottom quadrant represents the percentage of cells with reduction in ΔΨm. The data shown are representative of three individual experiments. (B) EGb761 induces activation of Bax and Bak. Mel-RM and Mel-AT cells treated with EGb761 (400 μg/ml) for the indicated intervals were subjected to measurement of activation of Bax and Bak by flow cytometry using antibodies that specifically recognize activated Bax and Bak, respectively. The filled and open histograms were generated from control (5% ethanol treated) (C) and EGb761-treated cells (E), respectively. The numbers represent MFIs. The data shown are representative of three individual experiments. (C) EGb761 induces mitochondrial release of cytochrome c and AIF. Mitochondrial and cytosolic fractions from Mel-RM and Mel-AT cells treated with EGb761 (400 μg/ml) for 16h were subjected to Western blot analysis. COX IV or GAPDH levels were included to show relative purity of the mitochondrial or cytosolic fractions. The data shown are representative of three individual experiments.

Bax and Bak oligomerization triggers loss of mitochondrial membrane potential (MMP)[38]. To study the involvement of Bax and Bak in induction of apoptosis by EGb761 in melanoma cells, we measured the MMP in melanoma cells treated with EGb761 for varying periods. Changes in MMP were monitored by staining with the fluorescent cationic dye JC-1 in flow cytometry [39]. In apoptotic cells, JC-1 transfers from mitochondria to the cytosol in monomer form and produce a cytosolic signal[40]. As shown in Fig 3A, EGb761-induced reduction in MMP that was detected at 16 h in Mel-AT cells. Consistent with relative resistance to EGb761-induced apoptosis, Mel-RM cells only displayed moderate reduction in MMP after treatment with EGb761.

Bax and Bak are activated by conformational changes upon treatment with a variety of stimuli, which can be detected in permeabilized cells using antibodies that recognize only the activated forms of Bax or Bak [41,42]. Fig 3B shows a marked increase in the levels of activated Bax and Bak in Mel-AT cells treated with EGb761. In contrast, activated Bax and Bak could hardly be detected in Mel-RM cells at 16 hours after treatment. These results suggest that the oligomerization of Bax and Bak and subsequent reduction in MMP are involved in apoptosis induced by EGb761, further confirming the role of the mitochondrial apoptotic pathway in EGb761-induced apoptosis in melanoma cells. In support, EGb761 triggered release of cytochrome C, Smac/DIABLO, and AIF from mitochondrial into the cytosol (Fig 3C).

### EGb761 disrupts the balance between anti- and pro-apoptosis Bcl-2 family proteins in melanoma cells

**Fig 4:**
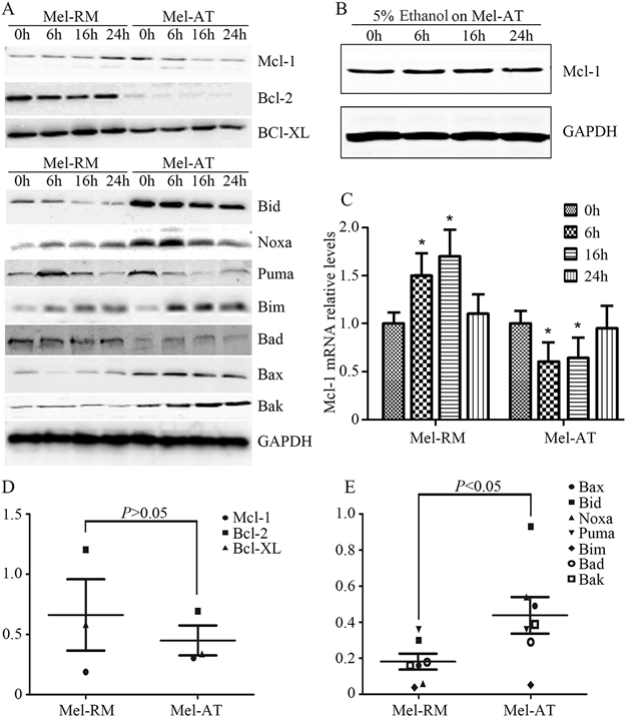
EGb761 regulates Bcl-2 family proteins expression in melanoma cells. (A) EGb761 alters the expression levels of anti- and pro-apoptotic Bcl-2 family proteins in melanoma cell lines. Whole cell lysates from Mel-RM and Mel-AT cells treated with EGb761 (400 μg/ml) for indicated time periods were subjected to Western blot analysis. The data shown are representative of three individual experiments. (B) 5% ethanol as control vehicle did not alter the expression levels of Mcl-1. Mel-AT cells with 5% ethanol for increasing periods. Whole cell lysates from Mel-AT cells treated were subjected to Western blot analysis. The data shown are representative of three individual experiments.(C) Mel-RM and Mel-AT cells were treated with EGb761 (400 μg/ml) or 5% ethanol for the indicated periods. Total RNA was isolated and subjected to real-time PCR analysis for Mcl-1. The relative abundance of mRNA expression treated with 5% ethanol was arbitrarily designated as 1. Columns, mean of three individual experiments; bars, SEM. * Present p<0.05 vs control.(D) Relative expression of anti-apoptosis Bcl-2 family proteins in melanoma cell lines Mel-RM and Mel-AT without treatment. Quantitative expression levels of Mcl-1, Bcl-2 and Bcl-X_L_ were normalized to GAPDH.(E) Relative expression of pro-apoptosis Bcl-2 family proteins in melanoma cell lines Mel-RM and Mel-AT without treatment. Quantitative expression levels of Bax, Bid, Noxa, PUMA, Bim, Bad and Bak were normalized to GAPDH.

The Bcl-2 family consists of a group of structurally related proteins that play a fundamental role in regulation of the intrinsic apoptotic pathway by controlling mitochondrial membrane permeability and the release of the pro-apoptotic factors [43]. The balance between pro- and anti-apoptotic Bcl-2 family members determines whether cells survive or commit to apoptosis. We hypothesize that EGb761 induced apoptosis by disturbing this balance in melanoma cells. To test this, we examined the expression levels of Bcl-2 family proteins by western blotting. As shown in Fig 4A and 4D, treatment with EGb761 resulted in different changes in the expression of the anti-apoptotic Bcl-2 family protein Mcl-1 in Mel-RM and Mel-AT cells. While EGb761 upregulated the levels of Mcl-1 in Mel-RM cells, it downregulated Mcl-1 in Mel-AT cells, which could be detected as early as 6 hours after treatment. By quantitation Mcl-1 mRNA levels, we found that the Mcl-1 transcript was increased in Mel-RM cells but decreased in Mel-AT cells by EGb761 (Fig 4C). Of note, treatment with the vehicle control (5% ethanol) did not alter the expression levels of Mcl-1 (Fig 4B). Although Bcl-2 was reduced by EGb761 in both Mel-RM and Mel-AT cells, its levels remained markedly higher in Mel-RM than Mel-AT cells after treatment. On the other hand, EGb761 did not induce any noticeable changes in the expression of the other pro-survival Bcl-2 family member Bcl-X_L_ in both Mel-RM and Mel-AT cells.

Noticeably, the pro-apoptotic Bcl-2 family proteins Bid, Puma, Bax, Bak and Noxa were expressed at higher levels in Mel-AT cells than in Mel-RM cells before treatment (Fig 4A and 4E). However, EGb761 similarly induced transient upregulation of Noxa followed by a decrease in both Mel-RM and Mel-AT cells (Fig 4A). In contrast, Bim was persistently increased by EGb761, whereas Puma was decreased in the cells after treatment with EGb761 (Fig 4A). Collectively, these results indicate that EGb761 disturbs the balance between pro- and anti-apoptosis Bcl-2 family proteins in melanoma cells.

### Mcl-1 plays a critical role in regulation sensitivity of melanoma cells to apoptosis induced by EGb761

**Fig 5:**
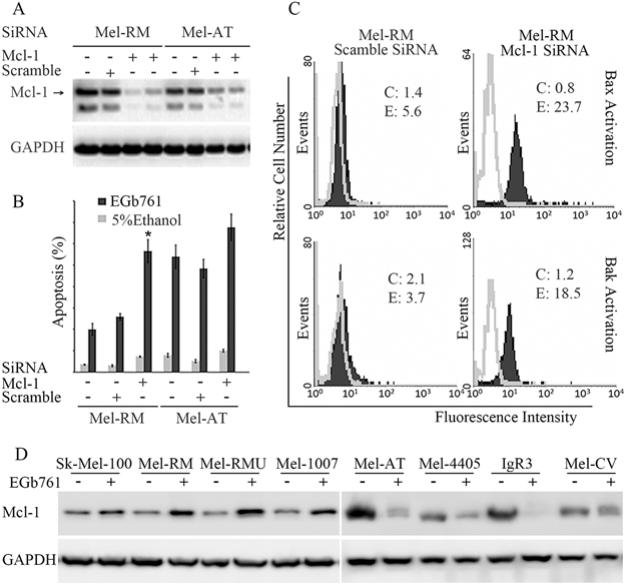
Mcl-1 plays critical roles in regulating the sensitivity of melanoma cells to apoptosis induced by EGb761. (A) Knockdown of Mcl-1by siRNA decreases the levels of Mcl-1expression. Mel-RM and Mel-AT cells were transfected with scramble SiRNA or Mcl-1siRNA. Whole cell lysates were then subjected to Western blot analysis.(B) Inhibition of Mcl-1 by siRNA induces apoptosis of melanoma and sensitizes melanoma cells to EGb761-induced apoptosis. Mel-RM and Mel-AT cells were transfected with scramble or Mcl-1siRNA. 24hours later, the cells were switched into normal culture medium for a further 48 h followed by treatment with 5% ethanol or EGb761. After another 24 hours, the cells were subjected to measurement of sub-G1content by the propidiumiodide method using flow cytometry. The data shown are the mean ± SEM of three individual experiments. * Present p<0.05 vs control.(C) Knockdown Mcl-1 can affect Bax and Bak activation status in Mel-RM cells. SiRNA inhibition of Mcl-1 resulted in a marked increase in the levels of conformational changed Bax and Bak, which detected by flow cytometry. The open and filled histograms were generated from control (5% ethanol) (C) and EGb761-treated (E) cells respectively. The numbers represent MFIs. The data shown are representative of three individual experiments.(D) Expression of Mcl-1 in a panel of melanoma cell lines after treatment with EGb761 or control (5% ethanol) for 24 hours. Whole cell lysates from melanoma cells were subjected to Western blot analysis. The data shown are representative of three individual experiments.

Since Mcl-1 was differentially regulated by EGb761 in Mel-RM and Mel-AT cells with different sensitivity to EGb761-inudced apoptosis, we further study the role of Mcl-1 in regulation of apoptosis triggered by the compound in melanoma cells by knocking down of Mcl-1 using siRNA. As shown in Fig 5A, siRNA knockdown of Mcl-1 reduced the levels of Mcl-1 expression by more than 80% in both Mel-RM and Mel-AT cells. Significantly, knockdown of Mcl-1 enhanced sensitivity of Mel-RM cells to EGb761-induced apoptosis (Fig 5B). The percentage of apoptotic cells after treatment with EGb761 was increased from 20% to 55% (Fig 5B). Moreover, knockdown of Mcl-1 also further enhanced induction of apoptosis by EGb761 in Mel-AT cells (Fig 5B). Associated with sensitization of melanoma cells to EGb761-induced apoptosis, knockdown of Mcl-1 promoted activation of Bax and Bak by EGb761 in Mel-RM cells (Fig 5C). The expressions of Mcl-1 were examined in a panel of melanoma cell lines with different sensitivity before and after treatment with EGb761 (Fig 5D). The results showed that EGb761 downregulated Mcl-1 in all the sensitive cell lines, but increased Mcl-1 in all the resistant cell lines. Taken together, these results indicate that Mcl-1 is a key regulator of apoptosis induced by EGb761 in melanoma cells.

## Discussion

Ginkgo has long been used in Oriental medicine. EGb761, a standard extract of the *Ginkgo biloba*, is widely used as a medicinal supplement [44], For example, it is used clinically in patients with cognitive function disorders, peripheral blood flow insufficiency, tinnitus, and vertigo [27,44]. Recently, its anti-proliferation and apoptosis-inducing effects on various cancer cells have been reported [27,45,46]. In a study performed in oral cavity cancer cells, EGb761 at 250 μg/mL induced caspase-3-dependent apoptosis. However, the underlying mechanism(s) remains poorly understood [47].

To better characterize the anti-cancer function of EGb761, we examined the apoptosis-inducing potential of EGb761 in melanoma cell lines. Our results showed that EGb761 induced varying degrees of apoptosis in melanoma cells, which did not appear to be associated with the mutational status of BRAF. In agreeing with previous reports, we found that EGb761 activated the caspase cascade, and that induction of apoptosis by the compound was markedly inhibited by a general caspase inhibitor. In addition, we found that EGb761 triggered activation of the mitochondrial apoptotic pathway and caspase-9 activation.

How does EGb761 activate the mitochondrial apoptotic pathway? Our study shows that apoptosis induced by EGb761 involved activation of Bax and Bak, which are essential for executing apoptosis in response to many apoptotic stimuli [48,49]. In normal cells, these proteins are distinctly localized [43]. While Bax is largely cytosolic and translocates onto mitochondria after apoptotic stimulation, Bak resides in the outer membrane of the mitochondria. Both proteins undergo conformational changes and homo-oligomerization in response to diverse apoptotic signals, leading to pore formation in the mitochondria outer membrane and release of the apoptosis-promoting proteins such as cytochrome c, Smac/DIABLO, and AIF [42]. Our results demonstrated that treatment with EGb761 resulted in conformational changes of Bax and Bak, which was associated with reduction in mitochondrial membrane potential in melanoma cells sensitive to EGb761-induced apoptosis. This is conceivably responsible for activation of the mitochondrial apoptotic pathway by EGb761 in melanoma cells.

The mechanisms of mitochondrial inducted cell death are usually related with p53 dependent or ER stress or direct interaction with mitochondrial proteins. Since EGb761 induced cell death in p53-null melanoma cells (ME4405), it suggested that induction of apoptosis by EGb761 is p53-independent. On the other hand, since EGb761 did not increase GRP78 and the cleaved form of XBP1, two commonly used marker of activation of the ER stress response, it is unlikely that EGb761 kills melanoma cell by induction of ER stress (data not shown). Instead, we found that EGb761 induced activation of the mitochondrial apoptosis pathway, which is associated with disturbance in the balance between pro- and anti-apoptosis Bcl-2 family proteins.

It is well-known that Bcl-2 family proteins are key regulators of apoptosis, in particular, in the case of involvement of the mitochondria apoptosis pathway [15]. To understand how EGb761 activate the pathway, we analyzed potential changes in the expression of Bcl-2 family proteins induced by the compound by Western Blotting. Of note there was no significant difference in the expression levels of the anti-apoptosis proteins Bcl-XL in sensitive (Mel-AT) and resistant (Mel-RM) melanoma cells before treatment. However, the levels of the pro-apoptosis proteins including Bid, Puma, Noxa, Bax and Bak appeared higher in sensitive compared with resistant melanoma cells. These pro-apoptosis proteins displayed similar changes in the expression after treatment with EGb761. These results suggest that the difference in sensitivity of melanoma cells to EGb761-induced apoptosis may not due to the difference in the changes of these pro-apoptosis proteins.

To further investigate the mechanism responsible for activation of the mitochondrial apoptotic pathway induced by EGb761 in melanoma cells, we examined whether EGb761 alters the expression levels of anti-apoptosis Bcl-2 family proteins. While both Bcl-2 and Bcl-XL did not appear to change in expression upon treatment with the compound, the expression levels of Mcl-1 were decreased in sensitive (Mel-AT) cells after exposure to EGb761 at transcriptional and translational levels. Further studies on a panel of melanoma cell lines demonstrated that EGb761 downregulated Mcl-1 in all the sensitive cell lines, but increased Mcl-1 in all the resistant cell lines. All above data suggest that Mcl-1 plays a unique apical role in regulating apoptosis, as elimination of Mcl-1 is required at an early stage for induction of apoptosis [19]. The significance of Mcl-1 in regulating EGb761-induced apoptosis of melanoma cells was further demonstrated by siRNA knockdown of Mcl-1. Indeed, inhibition of Mcl-1 by siRNA enhanced apoptosis induced by EGb761 in both sensitive and resistant melanoma cells. Mcl-1 is known to be critical for survival of melanoma cells under various stress conditions [19]. The expression of Mcl-1 is also known to increase in melanoma with disease progression [50].

In summary, we have shown in this study that EGb761 induces apoptosis of melanoma cells by disrupting the pro- and anti-apoptosis Bcl-2 family protein network. The imbalance between pro- and anti-apoptosis proteins of the Bcl-2 family plays a critical role in initiation of the mitochondrial apoptotic pathway [43]. Moreover, we have revealed that Mcl-1 has a major role in regulation of EGb761-induced apoptosis in melanoma cells. Since EGb761 is a standard extract containing 24% ginkgo layone glycoside including quercetin, kaenpferol and isorhamnetin, it is important to determine the specific component(s) of ginkgo layone that mediates induction of apoptosis by EGb761 in melanoma cells. Although further study is required in this regard, we have found that Quercetin alone induced apoptosis of melanoma cells (data not shown). Regardless, our results reveal that EGb761 induces apoptosis of melanoma cells through activation of the mitochondrial apoptotic pathway by disturbing the balance between pro- and anti-apoptosis Bcl-2 family proteins. Our results suggest that EGb761 with or without in combination with targeting Mcl-1 may be a useful strategy in the treatment of melanoma.
